# 5-Point programme for sustainable plant protection

**DOI:** 10.1186/s12302-018-0136-2

**Published:** 2018-03-14

**Authors:** Tobias Frische, Sina Egerer, Steffen Matezki, Christina Pickl, Jörn Wogram

**Affiliations:** Section Plant Protection Products, German Environment Agency (Umweltbundesamt, UBA), Woerlitzer Platz 1, 06844 Dessau-Roßlau, Germany

**Keywords:** Pesticides, Plant protection products (PPP), “Sustainable Use Directive” (2009/128/EC), Risk assessment, Risk management

## Abstract

This position paper intends to stimulate a profound rethinking of contemporary agricultural practice. We criticise the current intensity of chemical plant protection in Germany as ecologically unsustainable and thus threatening the achievement of key targets of environmental protection and nature conservation policies. In the first part of the paper, we provide background information on the use of plant protection products (PPP) in German agriculture, the role of agricultural policy, European pesticide legislation, the principles of and framework for environmental risk assessment and risk management of PPP, as well as environmental effects of PPP. The second part is presented against the backdrop of the European “Sustainable Use Directive” (2009/128/EC). This directive requires that “Member States shall adopt National Action Plans to set up their quantitative objectives, targets, measures, and timetables to reduce risks and impacts of pesticide use on human health and the environment and to encourage the development and introduction of integrated pest management and of alternative approaches or techniques to reduce dependency on the use of pesticides.” Reflecting on the corresponding debate in Germany, we suggest the following five key principles for a sustainable use of PPP and provide recommendations for their implementation: (1) minimising use; (2) identifying, quantifying, and communicating risks; (3) optimising risk management; (4) compensating for unavoidable effects; (5) internalising external costs.

## Background

### System dependence on chemical plant protection

#### Chemical plant protection in conventional crop production

**Fig. 1:**
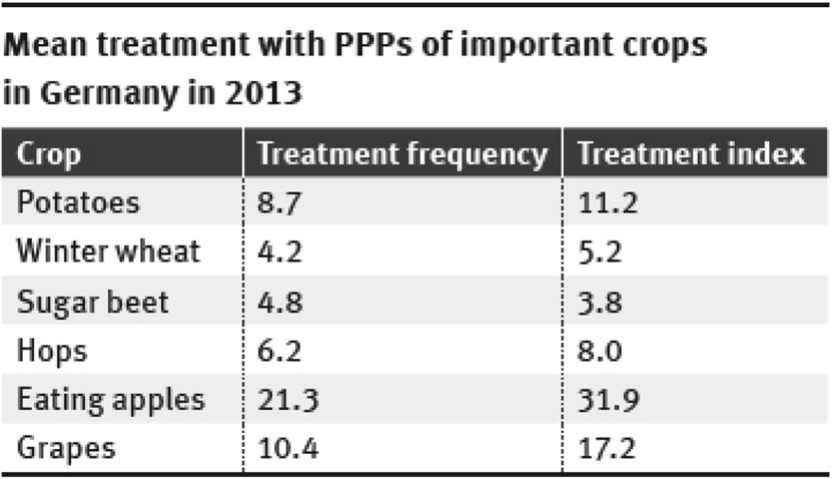
Mean treatment with PPPs of important crops in Germany in 2013 (our presentation, data from the PAPA Web site of JKI: http://papa.jki.bund.de/index.php?menuid=1, see also [5])

“Farming, forestry and agricultural business are among (…) the key sectors of the German economy…” [1]. This is true despite the fact that agriculture comprises only 0.9% to Germany’s gross domestic product (GDP) [2], since it is the crops cultivated by farms that provide basis for our existence. Cereals, fruit, and vegetables are among our most important foodstuffs, food crops feed our livestock, and for some years, we have grown increasing quantities of “energy crops” to produce biogas and electricity. This is not possible without the ample use of natural resources (land, soil, and water). About half the area of Germany (16.7 million hectares) is used for agriculture, and much of the German landscape has been shaped by crop production on some 285,000 farms [3]. Most farms (94%) operate the conventional crop production, which is characterised in particular by the use of mineral fertiliser and chemical plant protection products (PPPs). Mineral fertiliser provides maximum nutrient supply to the crops and the PPPs are used to tackle harmful bacteria and fungi, harmful animal organisms, and undesirable weeds. It is the combination of mineral fertiliser, chemical plant protection, and modern high yield crop varieties that make the current intensive crop production possible, with its tight crop rotation and monocultures. This in turn provides the high yields of conventionally produced plant products at consistently high marketable quality [4]. Since the “Green Revolution” in the mid-20th century, the conventional cultivation system has been continually optimised, so that it now provides the basis for our intensive agriculture and food production. The intensive use of chemical PPPs in conventional crop production is reflected in the annual surveys of the Julius Kühn Institute (JKI). Expressed as the so-called treatment index (i.e., number of PPPs used relative to the maximum permissible applied amounts and the cultivated area) in 2013 PPPs were applied an average of 4 times to wheat, 11 times to potatoes, 17 times to grape vine, and 32 times to apple trees (Fig. 1).

#### There is no natural law which dictates a “necessary minimum”

The current dominance of conventional farming systems is not only the result of free market forces but also of developments in German and European agricultural policies of past decades, which aimed mainly at increasing efficiency and yields [6]. European and national tax-funded subsidies for agriculture were a key factor in this context. Although now on the decline for several years, agricultural subsidies still account for some 42% of the total EU budget [2]. In Germany, the agricultural sector received a total of EUR 6.8 billion in 2012, and the average proportion of transfer payments in the income of German farmers was some 48% [2]. The European and national support for conventional cultivation systems not only offered advantages for farmers, but also for consumers. The reliable supply of high-quality fruit and vegetables and food products year-round at more and more favourable prices is something that consumers have come to expect. Ever higher consumer expectations (“unblemished and low-priced”) are also in part to blame for the current degree of dependence of the conventional crop production on chemical plant protection.

The notion of a “necessary minimum” (German: “notwendiges Maß”), which “denotes the amount of plant protection products that is necessary to secure the cultivation of crops, particularly from the aspect of economic viability” [7], has come to legitimise this dependence. This term, with its ideological undertones, suggests that the constraints of the market economy clearly leave the individual farmer with no choice other than the “necessary” use of PPPs. However, given the political influence on the market—in particular with farm payments—this line of argument is not entirely convincing. A different agricultural policy could well lead to a lower “necessary minimum”. However, implementing this would require political conviction (see below) while taking care not to lose sight of the reality of the global markets for agricultural produce.

(Note: The focus in the following is solely on PPPs. Relevant analyses and recommendations for mineral fertiliser (in particular nitrogen) are provided in other publications of the German Environment Agency (e.g., [8]).

### Assessment and management of environmental risks

#### Plant protection products: no effects without side effects

Chemical PPPs are used because of their biological efficacy in key areas: bactericides and fungicides are used to combat plant diseases caused by bacteria and fungi; insecticides kill insects such as aphids or caterpillars that damage plants; and herbicides are used to control “weeds”. To achieve these effects, the PPPs—which can consist of mixtures of up to 20 different chemicals—typically contain one or more chemically synthesised active substance. However, their effects are not usually very specific, i.e., not restricted to the target organisms in question. The description of the potential side effects is, therefore, an important element of the testing and approval procedure for PPPs. The direct effects of a PPP are described mainly on the basis of laboratory experiments in which indicator organisms such as algae, water fleas, fish, earthworms, bees, birds, and rats are exposed to the active substances or the PPP. These studies are used to determine the acute and/or chronic toxicity of PPPs for the so-called “non-target organisms”. Generally speaking, all PPPs can be expected to have more or less severe side effects—if the non-target organisms are exposed to relevant quantities. In other words: No effect (plant protection) without side effects (on organisms in the environment). The side effect profile of the PPP usually corresponds to the intended pesticidal effect: herbicides are particularly toxic for algae and non-target plants that are phylogenetic and biochemical relatives of “weeds”; similarly, insecticides are often just as toxic for beneficial insect species (honey bees, wild bees, butterflies, etc.) and other arthropods (spiders, woodlice, etc.) as they are for pest insects. With regard to bactericides and fungicides, the profile of the side effects is usually less clear. At the level of natural biocoenoses and ecosystems, it is known that direct PPP effects on certain organisms can lead in turn to indirect PPP effects on other organisms that are not directly affected by the toxicity (see further below).

#### Concerning risks and side effects ask the German Environment Agency

In view of their potential side effects and because they are introduced directly into the environment in considerable quantities and over large areas, the application of a PPP is only allowed in the European Union after it has successfully passed through a harmonised testing and approval procedure applicable in all Member States since 2012. In Germany, the legal framework is established by the Plant Protection Act [9] in combination with the European Regulation EC No. 1107/2009 concerning the placing of plant protection products on the market [10]. It requires an examination of the environmental impacts of each proposed application of a PPP to combat a defined harmful organism in a defined crop. The objective is not absolute protection or zero risk, but only that there should be no unacceptable effects on the environment. However, the risk assessment does not explicitly include weigh up the pros and cons of crop protection versus protection of the environment. Instead, it is carried out on the basis of legally binding decision criteria (at least for the standard assessment, i.e., lower tier) defined in the PPP Regulation [10] and in corresponding technical guidance documents.

In Germany, the German Environment Agency (UBA) is responsible for the environmental risk assessment of PPPs—including the impact on groundwater. The UBA commits considerable human resources to fulfilling this task with independent expertise and in accordance with the state of knowledge in science and technology. To ensure that the environmental impacts are acceptable the German Federal Office of Consumer Protection and Food Safety (BVL), which is the competent national body responsible for the approval procedure, draws on the risk assessment provided by UBA to formulate legally binding conditions of use and risk mitigation measures that are displayed on the PPP packaging and with which farmers have to comply. In the case of spraying this might involve technical regulations for the application (e.g., using drift-reducing technology) or requirements for maintaining a certain distance from surface water bodies. The list of PPPs currently authorised in Germany including the conditions of use and required risk mitigation measures can be accessed on the online BVL database (https://apps2.bvl.bund.de/psm/jsp/index.jsp). Monitoring compliance with the risk mitigation measures in Germany is the responsibility of the individual Federal States (*Bundesländer*).

### Residual risks, overall risks, and environmental impacts

#### Difficult predictions and residual risks

Even though UBA deemed the environmental impacts of each individual PPP authorised in Germany to be acceptable on the basis of current knowledge, residual risks remain that cannot be conclusively assessed. This is the case first for long-term risks, which in view of the complexity of the organisms and ecosystems can only be estimated to a limited extent and with considerable uncertainties through the available test and assessment methods. Gaps in knowledge will always exist despite any further scientific progress that can be taken into account in the official assessment procedure. The very complexity of the issue precludes certain knowledge and the ability to make predictions. The resulting residual risks of chemical plant protection are often overlooked in the public debate [11].

#### The total dose is what counts

**Fig. 2:**
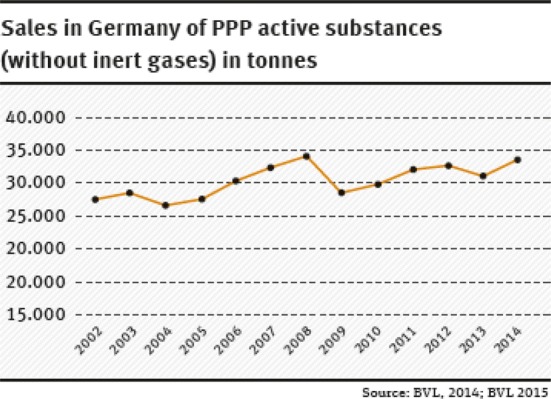
Sales in Germany of PPP active substances (without inert gases) in tonnes (our presentation, data taken from [12] and [13])

Another problem of the current authorisation procedure is that consideration of “the big picture” is omitted by examining the individual PPP application in isolation. As already seen with the treatment index, most crops are treated a number of times in the course of the growing season with the same and/or various PPPs. The sum of the applications and the total amount applied on a crop is, therefore, decisive for the overall risk or the environmental impacts in the agricultural landscape and not the individual PPP. A rough calculation demonstrates the general intensity of the use of PPPs in Germany: in 2014, 106,155 tonnes PPP containing 34,515 tonnes of active substances (without inert gases) were sold in Germany (BVL, 2015)—this amount has remained largely constant over the past 10 years or has even increased slightly (Fig. 2). Ignoring the differences in treatment intensity between crops, and assuming some 12.1 million hectares of arable land under cultivation, this gives an average application of 8.8 kg PPPs and 2.8 kg active substances per hectare.

#### When “theoretical” risks become real environmental impacts

**Fig. 3:**
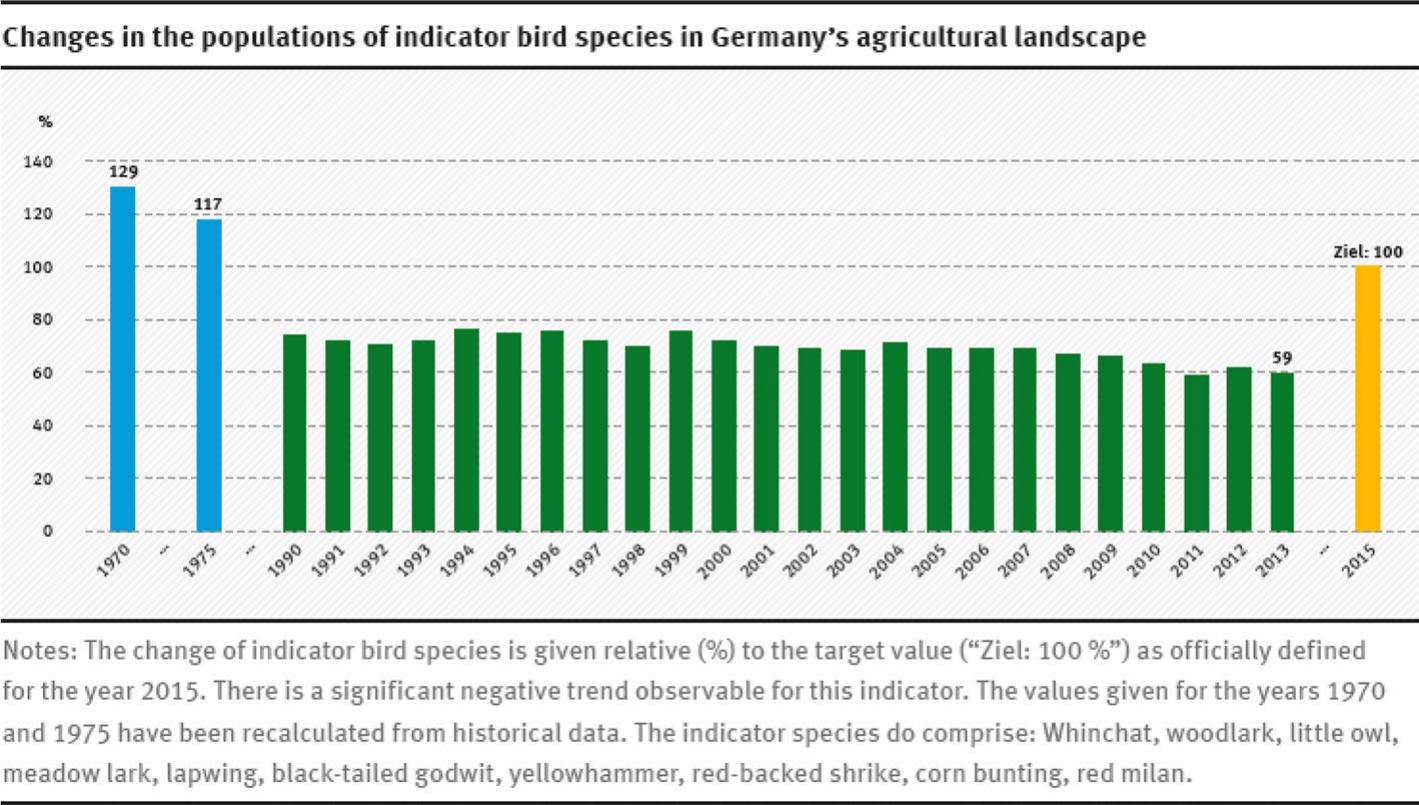
Changes in the populations of indicator bird species in Germany’s agricultural landscape (Figure copied from [23])
- *Tolyfluanid* The authorisation of PPPs containing this fungicidal active substance was withdrawn in 2007. The reason for this was the “delayed” discovery that a metabolite (*N*,*N*-dimethylsulfamide) which can find its way into groundwater, though previously classified as toxicologically harmless, can be transformed into a genotoxic and carcinogenic substance (*N*-nitrosodimethylamine) during the preparation of drinking water by ozonisation. The use of the active substance was prohibited as a precautionary measure for the protection of drinking water [24]. However, residues of other active substances such as atrazine, which has long been banned in Germany, are still found in groundwater and other currently authorised active substances (e.g., bentazon, isoproturon, and chloridazon), and their metabolites are frequently found in groundwater at concentrations above the limit levels [25]. There have recently been intensive debates in Germany about the pollution of groundwater and the problems faced by water utilities endeavouring to maintain the high quality of German drinking water. The position of the water utilities is “that the active substances in plant protection products and their metabolites should be kept away from the water cycle at the first opportunity for the sake of precaution” [26].

The remaining assessment uncertainties and the overall treatment intensity are more than mere “theoretical” risks: they have had and continue to have considerable negative impacts on nature and the environment. In retrospect, the development of chemical plant protection can be seen as an example of “pathological learning” [14]. It is now half a century, since the classic “Silent Spring” by Rachel Carson raised public awareness about the environmental damage caused by the first PPP generation [15]. The old active substances (DDT, organophosphates, etc.) have largely been replaced by more modern and much better tested active substances. However, three examples show the continuing relevance of PPP-environmental impacts:

- *Neonicotinoids* This group of highly effective insecticides have been widely used in the past 20 years for seed treatment (seed coatings). These “systemic” active substances are taken up by the seedling, offering protection against sucking insects and some chewing insects. In 2008, the spread of abrasion dust from coated seeds spread by pneumatic seed drilling equipment led to a massive poisoning of bee colonies in south-western Germany [16]. The importance of the airborne distribution of abrasion dust had been underestimated in the EU approval for the active substances and in PPP testing. The European Food Safety Authority (EFSA) reassessed the risk of the main neonicotinoids for honey bees and wild pollinators (e.g., bumble bees) in the light of the new scientific findings and found serious gaps in the data—in particular concerning long-term toxicity. For many applications, EFSA concluded that an unacceptable risk was indicated or could not be excluded. As a consequence, the EU Commission banned these critical applications in 2013 and called on the producers to provide the missing data [17]. It remains to be seen what decisions will be reached by the EFSA, the EU Commission and the national authorities on the basis of the data that is provided and in consideration of the massive criticism of this group of substances by scientists [18] and environmental and nature conservation associations (e.g., [19]).
- *Glyphosate* This is the most important herbicide in Germany and worldwide, and in contrast to neonicotinoids, it is currently thought to be relatively harmless for non-target organisms in the environment. The environmental problems arise from the blanket application of this broadband herbicide on a massive scale. The amount sold and used in Germany has risen sharply over the past 15 years and some 5000 tonnes are now used by German farmers every year [20]. This is due in particular to an increase in no-till cultivation. There are many environmental arguments in favour of less ploughing (e.g., reducing erosion on slopes, protecting against run-off from heavy soils, avoiding soil compaction, and improved soil water household), but in most cases, economic considerations are the main motivation for this trend. Farmers can save time and money if they control weeds using the relatively cost-effective glyphosate products. However, the massive use of glyphosate and other herbicides leads to a progressive loss of abundance and diversity in the farmland flora, with indirect effects on sensitive fauna. In the case of ground-nesting bird species such as the partridge, these food web effects are scientifically proven [21]. The elimination of field weeds by herbicides and of arable farmland insects by insecticides depletes food supplies such that the birds are not able to successfully reproduce in intensively farmed agricultural landscapes. As a result, populations are declining (Fig. 3). Chemical plant protection is one of the causes contributing to the disturbing continuous decline in biodiversity in the German agricultural landscape [22].

### Chemical plant protection and sustainability—a politically controversial topic

#### “Sustainable Use Directive” and the national action plan

In “A Thematic Strategy on the Sustainable Use of Pesticides” in 2006, the EU Commission had already noted that, in addition to the authorisation procedure, the use phase was decisive for the risks and impacts of PPPs [27]. As a consequence, EU Directive 2009/128/EC established a framework for Community action to achieve the sustainable use of pesticides, introducing “sustainability” as a political goal. The Directive obliges the Member States to draw up national action plans, “aimed at setting quantitative objectives, targets, measures, timetables, and indicators to reduce risks and impacts of pesticide use on human health and the environment and at encouraging the development and introduction of integrated pest management and of alternative approaches or techniques to reduce dependence on the use of pesticides […]” [28]. However, neither the Thematic Strategy nor the Directive proposed a precise quantitative reduction target for the application of PPPs. The European Commission, nevertheless, expected a significant reduction in PPP use as a result of the Thematic Strategy measures [27].

Some of the Directive requirements were transposed in Germany as “hard” regulations in the Plant Protection Act (e.g., regarding certificates of competence for sellers and professional users, or the aerial application of pesticides). In these cases, violations and infringements of key provisions of the law can lead to prosecution and the imposition of fines. However, most of the important requirements relating to nature conservation and environmental protection in the Directive were not transposed in the Plant Protection Act. However, they were included in the German National Action Plan on Sustainable Use of Plant Protection Products (NAP) adopted by the German Federal Government on 10 April 2013 [7]. The National Action Plan represents a comparatively soft regulatory instrument and most of the targets and measures it contains are not legally binding. The NAP is closer in character to a declaration of intent and the success of its implementation depends on the degree of motivation of the actors involved (in particular the Federal Government, Federal States, and agricultural associations) as well as on the funding available for implementation.

#### Sustainability in plant protection—controversy about the need for action

The German NAP was developed over a number of years in a multi-stakeholder process organised under the responsibility of the then Federal Ministry for Food, Agriculture, and Consumer Protection (BMELV, now Federal Ministry for Food and Agriculture, BMEL). The environmental protection and nature conservation associations, the professional beekeepers, and the water management sector were all critical of the draft version of the NAP. The associations voiced their criticism through termination of their further participation, stating in a press release dated 24.11.2011: “The Agriculture Ministry orients itself in the Action Plan towards the interests of the agricultural industry and seems deaf to suggestions to seriously reduce pesticide pollution. They will not receive support from the Associations for this.” [29].

Under the Plant Protection Act, UBA is involved in drawing up and implementing the German NAP in accordance with its responsibilities for the environmental risk assessment of PPP. In this context, UBA provided expert advice for the Federal Ministry for the Environment, Nature Conservation, and Nuclear Safety (BMU; meanwhile: Federal Ministry for the Environment, Nature Conservation, Building and Nuclear Safety, BMUB). The common goal of BMU and UBA was to realise an action plan that included targets and measures for environmental protection and nature conservation that were specific, binding, and ambitious. This was only partially successful, which is why UBA believes that there is a clear need for improvements with regard to environmental protection and nature conservation when the action plan is revised in the upcoming years (2016–2018). This view is not shared by the conventional agricultural sector where the general opinion is that plant protection in Germany is already sustainable. According to an EU Commission analysis, this attitude is widespread among the EU Member States: “The majority of NAPs appear to adopt the default position that the current PPP use pattern in their M(ember) S(tate) is sustainable.” [30]. This is confusing, because the Member States had in principle acknowledged the need for political action on the sustainable use of pesticides yet have so far seemed unwilling to follow through with any action.

## 5-Point programme for sustainable plant protection

UBA is of the opinion that the current intensity of the chemical plant protection in Germany is ecologically unsustainable and threatens the achievement of key targets of environmental protection and nature conservation policies. Plant protection that merits the attribute “sustainable” must be much more ambitious, specific, and transparent than is the case with the current German NAP. Reforms are also necessary with regard to the authorisation of plant protection products. To promote truly “sustainable development” in plant protection, UBA recommends an integrated approach for all relevant policy areas (plant protection, environment, nature conservation, and agriculture) based on the following five basic principles:

### Point 1: Minimising use

#### Anchoring minimisation in German plant protection legislation

From the perspective of nature conservation and environmental protection, the frequency of use of chemical PPPs and the amounts applied must be minimised. However, the farming sector is facing stiff competition, rationalisation pressure, and favourable prices for PPPs, with the costs of undesirable PPP impacts born by the public. With no effective incentives for farmers, it would appear the correct approach to introduce a legally anchored minimisation requirement. This would also start the urgently needed discussion among experts and policy makers about what actually constitutes a “necessary minimum” (“notwendiges Maß”) for the use of PPPs from a societal perspective. The requirement can be anchored in the “Code of good practice for plant protection” (Grundsätze für die Durchführung der guten fachlichen Praxis im Pflanzenschutz) [31], since the Plant Protection Act requires compliance with this code in every PPP use. However, three preconditions must be met for such a requirement to be fully effective:

i. If they are to use PPPs sparingly, farmers need better training and effective assistance from independent advisors about practical plant protection. In Germany, both tasks are now the responsibility of the plant protection services at the federal state level, but these are frequently understaffed [32]. As a result, advice on plant protection is predominantly given by consultants acting on behalf of the PPP producers and their primary goal is certainly not to advice on how to use PPPs sparingly. Therefore, widely available independent consultancy should be provided with the clear goal of “minimising PPP use”.
ii. An effective and independent monitoring system is required. It must be possible to determine whether an individual PPP user is, indeed, working to minimise the amounts applied, and checks must be carried out frequently enough to ensure effectiveness. The legal obligation of farmers to document their use of pesticides (in application logs) provides a suitable basis for traceability. The responsibility for checking compliance with the minimisation requirement could again lay with the plant protection services at the federal state level. Their remit would be to define criteria for good farming practice for plant protection in accordance with the minimisation requirement, taking into account the regional conditions and “pest pressure”, and to check compliance. This calls for random or targeted inspections of the application records.
iii. Obvious breaches of the minimisation requirement must be answered with appreciable sanctions. One option would be the reduction or withdrawal of Common Agricultural Policy (CAP) direct payments.

#### Integrated plant protection—back to the roots

**Fig. 4:**
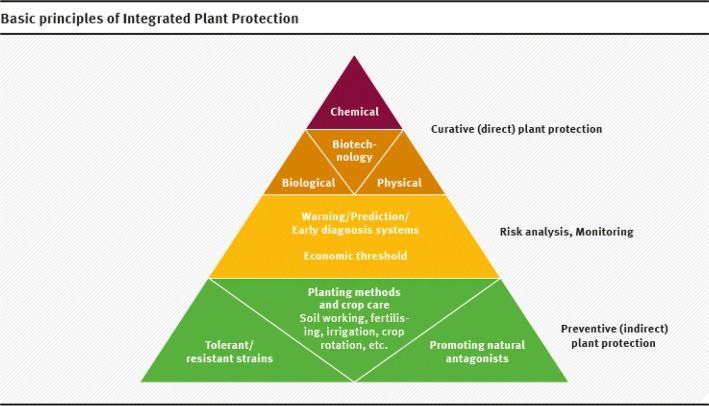
Basic principles of Integrated Plant Protection (from a talk on “Nutzen und Risiken von Pestiziden” by Dr. Eva Reinhard, BLW, Bern, 10.11.2014 at Oekotoxzentrum Dübendorf, Switzerland)

For conventional cultivation, “minimising use” means adopting an integrated approach to plant protection for which the basic principle is: “Chemicals are the last resort!” (Fig. 4). Integrated Plant Protection (often referred to as Integrated Pest Management, IPM) grants priority to preventative measures (choice of varieties, crop rotation, cultivation methods) and biological measures, in combination with the strict adherence to the economic threshold principle, before a chemical PPP is used [28, 33].

However, this “pure doctrine” has obviously slipped into the background, as can be seen from the increasing preventative use of pesticides (e.g., in the form of seed coating) and in uses not primarily relating to plant protection (e.g., herbicides to accelerate ripening and to kill off foliage before harvesting). For “real” Integrated Plant Protection, it is, therefore, essential to formulate specific minimisation requirements in the “Code of good practice for plant protection” and combine these with a consultancy and inspection system [34]. It is crucial that a universally applicable minimisation requirement does not justify any use of pesticides—for example on grounds of financial constraints and competition—which is not in accordance with Integrated Plant Protection (i.e., “The first to reduce spraying is the loser.”).

The implementation of the minimisation requirement in Integrated Plant Protection can be reinforced by setting up an agricultural equalisation fund—with the initial state support if applicable. The idea is to cushion the effects of loss of earnings or yield risks for the individual farmers who adopt plant protection methods aimed at minimising the use of pesticides. Relevant experience is already available from Italy [33]. In addition, increased support must be provided for research and development work on Integrated Plant Protection—in particular non-chemical methods.

#### Supporting the expansion of organic farming

The minimisation requirement must of course apply equally for plant protection in organic farming. Organic farming has already adopted these principles inasmuch as the use of chemical-synthetic PPPs is not permitted under the EC regulation on organic production and labelling of organic products [35], nor under the guidelines of the organic farming associations. Much smaller amounts of PPPs are used developed on the basis of natural substances (e.g., sulphur, copper, and pyrethrum), although this does not necessarily mean that they are not harmful to the environment. Organic farming, therefore, already meets the requirements of the EU “Sustainable Use Directive” 2009/128/EC for a cultivation system using smaller amounts of pesticides [28]. Another advantage is that an effective certification and monitoring system is already in place (EU organic logo and Germany’s eco-label as well as various association seals).

Increasing numbers of environmentally aware and health-conscious consumers approve of the goals of organic farming and place their trust in organic products, as can be seen by the steadily rising demand in recent years. Meanwhile, the demand for organically produced food in Germany exceeds the supply, thus requiring considerable amounts to be imported [36]. The fact that Germany’s organic farming lags behind demand is attributable to the current economic and agro-political climate which apparently makes the conventional crop production more profitable.

To promote organic farming as an effective way to reduce the environmental risks of and dependence on chemical PPPs, the framework conditions for those conventional farms that are willing to convert to organic farming must be improved. In particular, further training and assistance with conversion should be offered; improved financial support for organic farming is also needed. The Sustainability Strategy of the German Federal Government of 2000 set a target of 20% organic farmland by 2010, but it is currently still only slightly above 6% [3]. It is urgently necessary to review whether the increased incentives offered in recent times in some German Federal States (agro-environmental measures financed through the CAP “second pillar”) for converting to or staying with organic farming are sufficient to reach the target in the medium term. In 2014, the BMEL initiated the formulation of a “Strategy For The Future Of Organic Farming” [37] to provide more impetus for the expansion of organic farming in Germany. Together with representatives of the organic food sector and participants from the Federal States, science and various associations, the BMEL proposed strategies and recommendations for key fields which aim at achieving the target of “20% organic farming” anchored in the Sustainability Strategy of the German Federal Government (see also: http://www.ti.bund.de/de/thema/oekologischer-landbau/zukunftsstrategie-oekologischer-landbau/). The UBA welcomes this and calls for the vigorous expansion of organic farming in Germany, not least as an important element for sustainable plant protection. This endeavour will also require the strengthening of research and development for plant protection in organic farming.

#### Doing without chemicals in private gardens and public green spaces

The call to minimise the use of chemical PPPs also applies for public green spaces, private gardens and allotments. In contrast to farming, the economic benefits in this case are usually negligible and, accordingly, priority is placed on aesthetic considerations (“weed-free lawns”). Giving preference to non-chemical alternatives in these cases is, therefore, both practicable and reasonable. Information is already available on how to minimise the use of chemical pesticides in gardens and allotments (e.g., [38]), and further advice was quite recently released by UBA (http://www.umweltbundesamt.de/pflanzenschutz-im-garten-startseite). In addition to the voluntary avoidance of chemical plant protection, UBA also advocates a complete ban on herbicides in gardens and allotments. The background for this is the repeated occurrence of inputs of herbicides into public sewers and wastewater treatment systems, which in many cases is likely due to inappropriate application by private users, for example on paved areas [39, 40].

Regarding PPP use in public spaces, many German towns and local communities in the initiative “Pesticide Free Communities” (*Pestizidfreie Kommunen*) have committed themselves to avoiding the use of chemical PPPs either completely or to the greatest extent possible [41]. In France, a nationwide ban on chemical PPPs in public green spaces will come into effect from 2020 [42]. France has taken a big step towards implementing the measures under the EU “Sustainable Use Directive” 2009/128/EC to minimise or ban the use of pesticides in places such as public parks and gardens, playing fields and sports facilities, schools and children’s playgrounds, and in the vicinity of healthcare facilities [28].

#### Defining clear policy targets for the reduction of pesticide use

In view of the prospective revision of Germany’s NAP, UBA recommends a debate at policy-making level about a specific quantitative target for the reduction of the use of chemical PPPs. Germany would thus be following the examples of Denmark, which targeted a 40% reduction in PPP use from 2011 to 2015, and France, which has set specific reduction targets for 53 active substances [30]. A suitable starting point for the discussion about a pesticide use reduction target for conventional cultivation could be the experience gained in the “Demonstration farms for Integrated Plant Protection” (http://demo-ips.jki.bund.de/). Even under the current economic and agro-political conditions, they used up to 20% less PPPs than in comparable standard operating farms in the region as a result of expert counselling and adherence to the “threshold of damage” principle [43].

### Point 2: Identifying, quantifying, and communicating risks

#### Eliminating “blind spots” in the environmental risk assessment of PPPs

Being basically committed to the state-of-the-science, the authorisation process for PPPs demands that “blind spots” and weaknesses in the environmental risk assessment be eliminated. For example, the assessment currently fails to pay sufficient attention to the impacts on amphibians, reptiles, wild pollinators, soil arthropods, aquatic and soil fungi, or the indirect effects on biodiversity (for the latter, see also below). There is also considerable uncertainty about how representative the results of model calculations are of the PPP residues expected in the soil, groundwater, and surface water bodies. Improvement of the environmental risk assessment requires the continuous further development of the principles on which it is based as well as implementation of the scientific developments in appropriate testing requirements and assessment concepts. Responsibility for this development in Germany lies with UBA, in parallel to the processing of authorisation applications. Research projects commissioned by UBA for this purpose, the results of which are frequently introduced into the further development of the testing and assessment procedures at the European level. This revision process is initiated primarily by UBA for Germany and by EFSA with the goal of meeting the requirement to implement the state-of-the-art. Scientific progress is thus one of the reasons for the marked increase in the complexity of environmental risk assessment for PPPs in recent decades.

#### Critically reviewing “refined” risk assessment for individual PPPs

A further driver of the growing complexity of environmental risk assessment for PPPs lies in the EU Regulation on Plant Protection Products. In the event of a negative assessment result at a lower assessment tier based on standard data and conservative assumptions, the applicant can use a so-called “refined assessment” to show that no unacceptable environmental impacts of a PPP are to be expected under realistic application conditions. The investment in such a refinement of the risk assessment, e.g., in the form of mathematical models or more complex experimental studies (aquatic mesocosms, and field studies) is usually worthwhile for the applicant company. This is either because it is the only way to obtain an authorisation or because it makes it possible to avoid stricter requirements for risk management (e.g., concerning margins to adjoining surface water bodies). Refined risk assessment for environmentally critical but heatedly justified PPPs in particular is becoming increasingly complex.

This trend is questionable from a scientific point of view, because the more realistic risk assessment is still carried out in isolation for the individual PPP for which the application has been made. It ignores the fact that the exposure regime for the given crop will usually involve the multiple applications of various PPPs over the growing season. Increasingly, doubts are being expressed about the “refined” acceptability of the environmental impacts and whether the management requirements for an individual PPP are sufficient when the “total” exposure regime during a growing season is considered [44, 45]. The relevance of common forms and intensities of PPP application in tank mixtures and spraying with a series of PPPs compared to the evaluation and management of the environmental risks for individual PPPs has been and is being addressed in research projects commissioned by UBA (e.g., [46]).

Generally speaking, UBA sees the need to discuss the extent to which the trend towards increasingly refined risk assessment for individual PPPs (i) is appropriate or is as a rule disadvantageous for the environment, (ii) causes unnecessary societal costs, and (iii) places excessive demands on the risk communication (see further below). For an initial exchange on these questions, a working meeting initiated by UBA was held in November 2015, attended by experts from the relevant assessment authorities of a number of European countries.

#### Improving transparency and risk communication

Further objections raised against this “refinement trend” in risk assessment concern the increasing loss of transparency and greater susceptibility to the influence of special interests. Considerable scientific expertise is required for PPP producers to be able to further refine the risk assessment and for the assessment authority bodies to understand the measures involved. Quite frequently applicants do commission renowned scientists to prepare a scientific report for a refined assessment which is then submitted to defend the authorisation of the PPP or to argue that fewer management measures are required. Even if the assessment authority is able to respond with equal expertise (which becomes more difficult the smaller the agency of an EU Member State is), this development is problematic. In general, the more important the expert judgements become, the less transparent the decisions become for the public. The decision-making process becomes more susceptible to the influence of parties with special interests due to the relatively small numbers of actors involved. The independence of “super experts” and the democratic legitimation of their decisions, with their far-reaching implications, is a sensitive topic in modern knowledge societies [47, 48]. An indicator of the relevance and political sensitivity of this development has been the public criticism expressed regularly in recent years about the composition of EFSA’s panels of experts [49]. Many experts with earlier or existing connections to the chemical industry are believed to have conflicts of interests. To maintain or increase the public’s trust in the authorisation procedure for PPPs, the understandable call for the independence, transparency, and clarity of the decision-making processes must be answered. As a step in this direction, the German Federal Office of Consumer Protection and Food Safety (BVL) has been making public its authorisation decisions, including concise summaries of the evaluations, since 2009 (see http://www.bvl.bund.de). In the future, a significant step towards more transparency would be to make all the relevant data about the environmental behaviour and ecotoxicology of the active substance or PPP, including the results of confidential studies commissioned by the applicant, available in an open database. This is being considered both by UBA and EFSA.

#### Avoiding the complexity dilemma

As explained, the increasing complexity of the environmental risk assessment of PPPs is to some extent an unavoidable consequence of scientific progress. However, this does not apply for the complexity of the refined risk assessments for individual PPP enforced by applicant companies. In such cases, the principle of “risk management before risk refinement” offers a suitable way out of the looming complexity dilemma. For a PPP that could already be authorised, all justifiable options for the management of the environmental risks should first be exploited before the authorities approve and accept highly complex refinements of the risk assessment whose only aim is to create more favourable conditions for application of the PPP (e.g., narrower margins to adjacent surface waters).

A further regulatory alternative is offered by the “cut-off criteria” of the EU Regulation on Plant Protection Products [10], which provide for a ban on active substances with particularly hazardous properties, the so-called PBT substances [persistent (P), bio-accumulative (B), and toxic (T)]. A further exclusion criterion is targeting endocrine disruptors. The cut-off criteria represent a very progressive instrument in several pieces of Europe’s chemicals legislation, which is oriented more towards the precautionary principle than other international regulations. These criteria have been introduced by policy-makers and require a paradigm shift in the decision-making process. The decision on an approval or ban should in future only be based on the undesirable substance properties and not, as previously, on a quantitative risk assessment (i.e., the comparison of the expected environmental exposure concentrations with (eco)toxicological threshold concentrations/doses for the harmful impacts on non-target organisms). Experts justify the hazard-based regulation by referring to the high level of uncertainty in the risk assessment for the targeted hazardous properties. The cut-off criteria provide an impulse to develop and use active substances and PPPs that have lower environmental impacts. However, a practical implementation is currently not possible, because the scientific and technical details of the cut-off criteria have not yet been developed and specified in subordinate regulations. The delays in implementation have been due not least to the massive interventions of the European PPP industry, which categorically rejects hazard-based regulation and demands a return to risk assessment, even for undesirable substance properties [50]. The UBA believes that the cut-off criteria are in principle well suited to improve the protection of the environment against particularly hazardous PPPs. Because of this conviction and in accordance with its responsibilities, UBA is also involved in the discussion about the specification and implementation of the cut-off criteria [51, 52]. One particular challenge is that it is not always clear from an environmental point of view whether an alternative active substance really is better than the banned active substance it is meant to replace. The same task of comparative assessment applies for PPP approval: The EU regulation provides that a PPP containing so-called substitution candidates (e.g., active substances with two out of three PBT properties) should be replaced by a PPP with a lower environmental impact. The methodology of comparative assessment of the environmental hazard or risks of PPPs is—both from a scientific and regulatory viewpoint—challenging and has not been sufficiently tested so far [53]. However, the general public and users rightly demand that in future the assessment authorities should make increased use of their expertise to provide information about the more environmentally favourable alternatives. It remains to be seen how effective the instrument of comparative assessment will prove in terms of making environmental impacts measurable.

#### Describing the overall risks and impacts of PPPs

A further challenge is the description of the environmental risks and environmental impacts that result from the overall intensity of chemical plant protection in Germany. There is a need for scientifically relevant indicators that are understood by the public and can be used for their information and for policy-making. Some environmental indicators were used to review the progress of the German NAP. Comparable to the risk assessment for individual PPPs, the SYNOPS indicator calculates a generic risk index for plant protection intensity in Germany for selected non-target organisms, e.g., for aquatic and soil organisms, and bees [54]. In addition to this “theoretical” SYNOPS risk, the German NAP also includes indicators for the actual environmental status (e.g., pollution of surface water bodies by PPP residues, development trends of bird species populations in the agricultural landscape). Ideally, data from environmental measurements and environmental monitoring allow conclusions to be drawn about: (i) the plausibility of the risk assessment in the approval procedure, (ii) the efficacy of PPP-specific environmental risk management, and (iii) changes to the environmental status through the general use trend of PPPs. At present, there is no representative PPP-specific monitoring in Germany for all potentially affected environmental compartments, ecosystems, and organisms. The German NAP only collates existing monitoring programmes, giving an incomplete overview of the current environmental impacts of chemical plant protection. The UBA sees a clear need for improvements and has commissioned an on-going research project within the framework of the German NAP to develop of a strategy for monitoring the pesticide loads of small surface water bodies in the agricultural landscape [55]. The small surface water bodies make up a large proportion of the overall network of surface waters and are at greatest risk of pollution with PPPs due to their proximity to the application areas. However, they are currently underrepresented in the monitoring scheme pursuant to the EU Water Framework Directive. The UBA also initiated a research project to test whether and how integrated monitoring could improve the description of the environmental impacts of chemical plant protection. Integrated monitoring records both the fate and exposure of PPPs or PPP residues in the environment as well as their impacts on organisms, ecosystems, and ecological processes. This parallel registration is necessary to be able to identify the specific contribution of the chemical plant protection to changes in the environmental status, in particular if the processes are influenced by a number of different factors (e.g., the changes in population of bird or amphibian species in the agricultural landscape).

### Point 3: Optimising risk management

#### Limiting PPP applications in protected areas

The simplest and most effective way to avoid the risks and impacts of chemical PPPs is not to use them. UBA believes that PPPs should not be applied on public green spaces, private gardens, and allotments, nor—wherever possible—also in nature conservation and drinking water protection areas. This recommendation is in accordance with the EU Sustainable Use Directive, which stipulates a minimisation of or ban on the use of PPPs for nature conservation areas (protection areas for birds, FFH areas) and drinking water protection areas [28]. However, this is not transposed into a national regulation in Germany; rather the Plant Protection Act passes on the responsibility for introducing the appropriate measures to the Federal States (Article 22 of [9]). As in an information paper on emergency approval for the use of PPPs in nature conservation areas [56] which was published by UBA and the German Federal Agency for Nature Conservation (BfN), UBA also urges the Federal States to put in place a general ban on the use of PPPs in these areas.

#### Minimising distribution in the environment with modern application technology

If the use of chemical PPPs is unavoidable, they should be applied making the best possible use of the available technology and economically justifiable options for risk management. The goal is to stop PPP residues—to the extent possible—from entering into or spreading to non-target areas, natural assets (e.g., groundwater and surface waters) and habitats adjacent to the application areas. Although this will never be completely feasible, further improvements are possible through technical risk management. Technology should ensure that the PPPs are applied as accurately as possible and without losses and spillage—whether they are in solid form (seed coating and granulates) or in liquid form (for spraying). In contrast, aerial applications (e.g., by helicopter) are difficult to control and are, therefore, generally banned in Germany under the Plant Protection Act. There are a few exceptions, for example treating the crown zone of forests or steep vineyard slopes [9, 56]. When applying PPPs on arable land and for special crops (fruits, vines, and hops), it is usually necessary to use mobile spraying gear with drift-reducing nozzle technology. Introducing the best available nozzle technologies is thus an effective way to reduce environmental pollution by PPP residues. The German NAP formulates the same target but without specifying any measures to be adopted. Possible options include the introduction of an appropriate innovation and subsidy programme or granting tax credits for the adoption of modern technology.

#### Effectively monitoring compliance with legal risk mitigation measures

Farmers who fail to comply with the legally binding PPP-specific risk mitigation measures for the protection of the environment are subject to fines. Key requirements concern the maintenance of untreated margins of fields adjacent to bodies of water and terrestrial habitats (e.g., marginal biotopes and forest margins). As a rule, however, the yield and crop quality is lower for these untreated areas of farmland, resulting in financial losses as a consequence of compliance with result from observing the spacing requirements [57]. Just as road speed limits tend to be ignored if there are no regular speed checks, there is a risk here too that without regular checks and the prosecution of transgressions, these “inconvenient” regulations will lose their effectiveness and the number of transgressions will increase. However, the extent to which plant protection products are applied in accordance with the regulations in Germany is unclear. The results of the checks carried out by the Federal States are documented in annual reports on the plant protection monitoring programme (www.bvl.bund.de/psmkontrollprogramm), but these do not provide a basis for drawing conclusions. The 2013 report shows that relatively few checks were carried out. Compliance with the spacing requirements for the protection of surface water bodies was only checked for 423 application areas of 421 agricultural holdings in Germany—less than 1% of all German farms. The main reason for this is the understaffing of the Federal States plant protection services. However, the results from 2013 also point to non-compliance with legal requirements: Transgressions were identified in 10% of the inspections, but the report does not specify whether these were intentional or due to a lack of relevant knowledge. Intentional breaches of the legal requirements must be countered with increased controls and the penalisation of all transgressions. However, if a lack of specialist knowledge is the root cause, then this calls for a review of the further training courses offered for professional PPP users seeking to obtain the legally required certificate of competence. A central module of the curriculum should explain the importance of nature conservation and environmental protection and the obligation to comply with the relevant legal stipulations.

#### Reducing PPP risks through landscape management

In the opinion of UBA, additional risk management options should be implemented that are as independent from the behaviour of individual PPP users as possible. Agricultural landscape management is an effective measure as it would at the same time simplify risk management and in part make it unnecessary to monitor compliance with risk mitigation measures (here: spraying distances). The basic idea is to separate the treated area from the adjacent environment. By establishing permanent green margins and buffer strips or permanent three-dimensional vegetation structures (e.g., hedges, waterside margins with bushes, and trees), the airborne transmission and run-off of PPPs to adjacent non-target areas or bodies of water is avoided or at least considerably reduced. Switzerland is a role model in Europe in this regard, requiring 3 or 6 m-wide green buffer zones along surface waters [58]. A similar regulation has also been in place in Denmark since 2012 [59].

The NAP has set a long-term target for Germany to create buffer zones, permanently covered with vegetation and at least 5 m in width, for all surface waters in the agricultural landscape. However, no timeline is specified in the action plan, and the implementation is the responsibility of the individual Federal States (e.g., by including support for the creation of waterside margins in agro-environmental programmes). Some Federal States have already initiated appropriate measures [60], but there is currently no systematic overview of the progress made in creating permanent green waterside margins for Germany as a whole. The German NAP has set an ambitious target for the creation of buffer zones by 2023 for all surface waters in protected areas for drinking water, nature reserves, and in sensitive areas identified by hot-spot analyses. There is general consensus that the greening requirement should be applicable which is valid since 2015 under the EU Common Agricultural Policy (CAP) for such landscape-based risk management. To receive the full CAP area-based payments, farmers are required to dedicate 5% of arable land to ‘ecological focus areas’. The NAP Forum (December 2014) concluded: “The NAP Forum is of the opinion that the primary use of ‘ecological focus areas’ to create buffer strips, field margins and forest peripheries where the application of PPPs is banned under the Greening rules can provide an important contribution for the protection of surface waters and the preservation of biodiversity by increasing the proportion of habitats and sanctuaries in the agricultural landscape.” [61]. UBA expressly supports this recommendation and argues for the implementation of this effective approach to PPP risk management optimisation in Germany as widely and as quickly as possible.

### Point 4: Compensating for unavoidable effects

#### Taking indirect effects of PPPs on biological diversity into account in environmental risk assessment

As already explained for the example of glyphosate, the indirect effects of the use of chemical PPPs are one of the factors responsible for the decline of biological diversity in the German agricultural landscape [22]. Indirect effects arise, because the intended rigorous elimination of the field weeds by herbicides and of farmland insects by insecticides also leads to a reduction in the food supplies for wild animals. As a result, they are unable to reproduce successfully and their populations decline. In the past, such effects on food webs and habitats were ignored or their relevance was underestimated, despite the fact that the EC Plant Protection Products Regulation (EC No. 1107/2009) expressly calls for impacts on biodiversity to be taken into consideration for the approval of PPPs [10]. However, there are not yet any harmonised methods at the EU level to assess the indirect impacts of PPPs on biodiversity.

#### Using ecological focus areas for risk management

Risk management must be improved promptly if the legal requirement for the protection of biodiversity from the indirect effects of PPPs is to be met. UBA recommends the introduction of special risk mitigation measures: Prerequisite for the use of PPPs with a high risk of indirect effects on biodiversity should be the existence of ecological compensation areas where PPPs are not applied at farm level. Such compensation areas could include set-aside areas, flowering margins, and untreated thinly sown areas. This landscape-related requirement aims at a compensatory reduction of risk. The ecological compensation areas should offset the unavoidable direct effects of the PPPs on the treated areas to the extent that they are reduced to an acceptable level. The ecological compensation areas should offer typical farmland fauna at least the space needed for foraging and retreat.

#### Introducing PPP-specific risk mitigation measures for authorisation

This new application requirement should be included in the approval procedure using a risk-based approach. This means that the requirement should be imposed not as a blanket measure for all PPPs but based on the risk assessment for the individual PPP. Such a requirement should only be imposed for PPPs with a high risk of indirect impacts on biodiversity. Each PPP would have to be examined as to whether the application in question would reduce food organisms or plants on the treated areas, such that their habitat function for higher organisms is impaired (in particular birds and mammals). This assessment can be conducted quantitatively on the basis of the existing data. Based on a preliminary estimate, a large number of PPPs would be affected by the new requirement (nearly all herbicides and insecticides and about a third of fungicides). However, this is not particularly relevant for conventional holdings or those which have integrated measures, because the application requirements will be the same for all the affected PPPs. Once a farm has complied with these requirements, it will be able to use all PPPs. The UBA also favours the introduction of such new risk mitigation measures initially only for field crops and primarily for those regions with a high proportion of land used for agricultural purposes (i.e., “agricultural steppes”) which have particularly poor “ecological infrastructure” (hedges, waterside buffer zones, forest margins, field verges, and extensive grassland) in terms of the protection of biodiversity as a consequence of rural restructuring. The UBA and the German Federal Office of Consumer Protection and Food Safety (BVL) have discussed the need for compensation measures to protect biodiversity against the indirect impacts of PPPs. Whereas a concept for their implementation has been drafted important questions of detail remain to be clarified concerning the lack of a standardized assessment method in the EU, legally valid evidence of a PPP’s potential to harm biodiversity, and methods to ensure compliance. Another issue is the necessary minimum proportion of ecological compensation areas at the level of the individual holdings. The UBA sees that while taking economic viability into consideration, the ecological compensation areas without PPP application should comprise no less than 10% of the cultivated area of a holding. This proportion has already been shown to be reasonable, because there was a minimum set-aside quota of 10% in the EU until 2006 [21].

#### Insisting on a contribution from PPP risk management to protect biodiversity

The UBA’s thoughts regarding compensation measures for the indirect effects of PPPs on biodiversity have been vehemently rejected by the conventional farming associations and PPP producers. They draw attention in a joint position paper to the fact that the greening requirements of the EU Common Agricultural Policy (CAP) also serve to protect biodiversity, e.g., the requirement for 5% ecological focus areas. They also argue that “the protection of biodiversity is already addressed in a variety of ways by the environmental and agricultural policies.” [62]. However, neither the dedication of 5% of arable land as ecological focus areas in accordance with the CAP greening, nor the agro-environment measures from the CAP “second pillar” are sufficient to protect biodiversity in landscapes which are heavily affected by agriculture, as is required under German plant protection legislation ([63], see also: 21). It is true that the ecological focus areas in accordance with CAP can also be effective for the proposed PPP-specific application requirement for the protection of biodiversity. Therefore, the UBA supports the corresponding recommendation of the NAP Forum from December 2014 (see further above).

Despite the critical reactions, the UBA is convinced that its proposal can provide an important contribution to the implementation of the German National Strategy on Biological Diversity [64]. This includes the following target: “By 2015, the populations of most species typical of agriculturally cultivated landscapes will have been secured and will have begun to increase again.” Effective action is now urgently needed as this goal has not yet been achieved, in part due to the growing pressure on farmland which among others is due to the increased cultivation of energy crops and fodder crops for intensive livestock farming [22]. The chemical plant protection sector as a significant player must also make a contribution, not least in its own interests. Public confidence in the possibility of a plant protection that is compatible with nature and the environment should not be further endangered, and trust should be restored, even if this means acknowledging the necessity of self-limitation and a future with less intensive use of PPPs.

#### Learning from models for biodiversity conservation in conventional agriculture

The Swiss production label “IP-Suisse” (http://www.ipsuisse.ch) is a model for the successful implementation of voluntary measures to protect and promote biodiversity in the conventional crop production. Certified farms adopt various measures to promote biodiversity (e.g., lark nesting gaps, multi-year fallow, extensively used grassland, planting hedgerows, etc.). Compliance with the biodiversity requirements of the IP-Suisse guidelines is regularly assessed according to a points-based system. A network of advisers supports the farmers with the planning and implementation of measures to promote biodiversity. In addition to area-based compensatory measures, there is also a marked reduction in the use of PPPs for various crops, e.g., cereal crops are free of growth regulators, fungicides, and insecticides. This is economically viable, first, because less vulnerable varieties are used, and second, because higher prices can be charged under the IP-Suisse label.

### Point 5: Internalising external costs

#### Paying more attention to the social dimension of sustainability

Private sector activities may, in many cases, generate macroeconomic benefits, but they can also result in costs for the general public. Ideally, sustainability should involve a just distribution of the benefits and costs of commercial activities so as to maximise the common good—for today’s society and for future generations. Whether current chemical plant protection meets this requirement is a topic of heated debate. The central questions are: Do the social benefits outweigh the social costs? Are the benefits and costs distributed fairly between the relevant stakeholders (PPP producers, farmers, trade, consumers) and the affected parties (all citizens, tax payers, and future generations)?

#### Raising awareness about the “external” social costs of chemical plant protection

As described in the introductory chapter, the use of chemical PPPs offers clear short-term benefits for the farmers (high, stable yields, and marketable quality) and also for consumers (secure supplies and low shop prices). The producers, suppliers, and users of PPPs regularly point out that in addition to the directly measurable benefits for agricultural operators, chemical plant protection also provides considerable macroeconomic benefits. A study commissioned by the German Agricultural Industry Association (IVA) emphasises the “special role of plant protection for specific socially relevant objectives” and estimates the overall annual benefit of chemical plant protection for society at between one and four billion euros [65]. However, a crucial weakness of this study is that it only takes into account macroeconomic benefits without considering the societal costs. The “positive welfare effects” of chemical plant protection identified by the authors should be contrasted with the negative external impacts and costs to provide a more complete picture. These costs are borne by the whole of society (“socialised” costs) for the necessary monitoring and testing apparatus as well as for the impacts on human health and the environment. These costs are “external”, because they are not fully reflected in the market prices of the plant protection products, the harvested crops, and foodstuffs. This cost externalisation is cited as one of the main reasons why the retail prices for conventionally produced food are much lower than prices for organic food products [66]. Most consumers are not aware when they choose lower cost, conventionally produced goods that they ultimately pay an indirect price which is considerably more than the price at the cash till. This is explained partly by farm subsidies funded through taxation and in part to the externalised costs of conventional cultivation systems. The societal costs will either have to be met now or may be borne by future generations who have not had none of the current benefits.

#### Various types of external costs have to be taken into account

The main external effects or costs to be taken into account for a comprehensive analysis include in particular:

- Survey, monitoring, and repair costs:
  - For PPP residues in groundwater and surface waters: costs incurred for monitoring, avoidance measures, and for water treatment are borne by the relevant authorities, by water suppliers and their customers.
  - For PPP residues in agricultural produce or processed food commodities. The costs for monitoring levels of residues are incurred for the official monitoring programmes and for the extensive testing in food retailing. Official testing is tax-funded, whereas the food industry passes the costs on to the consumer.
  - Other official monitoring costs that are not fully passed on to the PPP authorisation holder or PPP users in the form of fees and are instead tax-funded (e.g., the portion of costs for official authorisation procedures that are not refinanced, consultancy, and monitoring costs of the plant protection services of the Federal States, costs of PPP-specific research by public research institutions).
- Health costs as a result of acute or chronic exposure of PPP users, local residents, or third parties and consumers to PPPs or their residues. These include costs for medical treatment, lost working time, and the non-material costs of health impairments (suffering).
- Costs for agricultural production:
  - Direct costs (bee-keeping, honey production) and indirect costs (pollination services) as a result of acute or chronic pollution of honey bees with PPP residues.
  - Costs for the impairment of ecosystem services, e.g., natural biological plant protection by beneficial insects, pollination by wild pollinators (e.g., bumble bees and solitary bees), or the production function of soil by soil organisms (e.g., earthworms).
- Costs of impacts on nature and the environment:
  - Impacts on aquatic organisms and the biodiversity of surface water bodies by PPP residues as a result of accidents, inappropriate use, or unavoidable diffuse inputs (dust or spray drift).
  - Impacts on biodiversity in soil as a result of unavoidable PPP inputs in the soil.
  - Impacts on the biodiversity of wild plants and invertebrates (insects, spiders, etc.) in the agricultural landscape as a result of diffuse inputs of PPPs (via dust or spray drift) in habitats adjacent to the treated areas.
  - Direct effects of PPP applications (acute or chronic poisoning) and indirect impacts (food web) on vertebrates (birds, mammals, amphibians, reptiles, and fish) and the biodiversity of vertebrates in the agricultural landscape.

#### Tackling the methodological challenges

For various reasons, the quantification of the costs of impacts on nature and the environment represents a considerable challenge. First, the methodology for monetary assessment of environmental impacts is still in its early stages. This is not surprising, because it involves fundamental questions that cannot be answered objectively (e.g., “What is the value of a partridge?”). Second, a suitable database is often lacking for the assessment of environmental costs. This is the case in particular for the description or quantification of the specific/relative contributions of chemical plant protection to adverse impacts or general trends observable in the environment, e.g., effects on water organisms of pollution with both PPP residues and nutrients. Currently, the data and analyses needed for a rational and fact-based discussion are not available. The only comprehensive, independent cost–benefit analysis for Germany was commissioned in 1991 by the Agriculture Ministry [67]. This study still has a model character, because no analyses of comparable scope have since been carried out in Germany. Meanwhile, however, conventions and proposals regarding the criteria for socio-economic assessments of environmental impacts have been developed, e.g., at the international level [68] and also by UBA [69].

Finally, monetary assessment also raises fundamental ethical questions. For example, would humanity be justified in allowing the extermination of individual animal and/or plant species if this provided an economic benefit?

#### Discussing the need for political action on the basis of sound data

The UBA sees a need for a systematic review of and a political discussion about both the external costs of chemical plant protection in Germany and the distribution of the costs within society. UBA commissioned a study drawing on the work of Waibel and Fleischer [67] to provide scientific clarity. In a second step, it is necessary to discuss the scope for political actions concerning the “societal dimension” of chemical plant protection. This should also address the potential options for political action to compensate for the effects of market distortions and to internalise external costs. Political influence could be exerted by means of reforms to the EU and national farm payments (e.g., increased payments for farms with low PPP use) or a levy on PPPs (which is common practice in some EU Member States, e.g., in Denmark). A study on the introduction of a levy or tax on PPPs in Germany carried out on behalf of the Federal States Schleswig-Holstein, Baden-Wurttemberg, and Rhineland-Palatinate recently reignited a discussion on this topic [70]. The UBA expressly welcomes the discussion about the prospects and the limits of this instrument—both with regard to the internalisation of external costs and to providing incentives to minimise the use of chemical plant protection products.

## Abbreviations

BfN: German Federal Agency for Nature Conservation
BMEL: German Federal Ministry for Food and Agriculture
BMUB: German Federal Ministry for the Environment, Nature Conservation, Building and Nuclear Safety
BVL: German Federal Office of Consumer Protection and Food Safety
CAP: Common Agricultural Policy (EU)
EFSA: European Food Safety Authority
EU: European Union
IPM: Integrated Plant Protection (often also referred to as Integrated Pest Management)
IVA: German Agricultural Industry Association
JKI: Julius Kühn Institute (German Federal Research Centre for Cultivated Plants)
NAP: German National Action Plan on Sustainable Use of Plant Protection Products
PPP: Plant Protection Products
UBA: German Environment Agency (Umweltbundesamt)
